# Terahertz Enhanced Sensing of Uric Acid Based on Metallic Slot Array Metamaterial

**DOI:** 10.3390/mi13111902

**Published:** 2022-11-03

**Authors:** Yuke Han, Xiaomeng Bian, Misheng Liang, Tianshu Li, Lianqing Zhu, Xiaoguang Zhao, Rui You

**Affiliations:** 1 Key Laboratory of the Ministry of Education for Optoelectronic Measurement Technology and Instrument, Beijing Information Science and Technology University, Beijing 100015, China; 2 Beijing Advanced Innovation Center for Integrated Circuits, Beijing 100084, China; 3 Department of Precision Instrument, Tsinghua University, Beijing 100084, China

**Keywords:** uric acid detection, terahertz metamaterial, biosensor, enzyme-free detection

## Abstract

An enzyme-free terahertz uric acid sensor based on a metallic slot array metamaterial was proposed and realized both theoretically and experimentally. The sensing model was verified in simulation and femtosecond laser processing technology was employed to ablate slots in the copper plate to fabricate metamaterials. Analytes were tested with liquid phase deposition on the metamaterial by a terahertz frequency domain spectroscopy system. Gradient concentrations of uric acid, ascorbic acid, and a mixture of them were measured separately with a good linear response. A significant decrease in sensitivity was observed in the ascorbic acid assay compared with the uric acid assay. The test results of the mixture also proved that our device is resistant to ascorbic acid. It is a simple and effective method for monitoring uric acid concentrations and the strategy of eliminating interference while modulating the resonance peak location mentioned here can be rationally projected for the development of other sensors.

## 1. Introduction

Hyperuricemia, a metabolic disease like diabetes, is rising owing to modern human dietary changes [1,2]. It shows typical chronic disease features, like mild initial symptoms, progressive deterioration of health, and complex complications in the later period [2,3,4]. The global hyperuricemia incidence rate is now around 5~7%, indicating a huge influence on the health and economy of society [2]. A specific clinical symptom of hyperuricemia is long-standing excess uric acid (UA) level (≥420 μM) in the blood, hence the necessity of blood UA monitoring with high efficiency for both diagnostics and therapeutics.

Great efforts have been put towards developing methods of monitoring UA academically, including spectrophotometry, mass spectrometry, and chemical sensors based on electrochemical methods, among others. Enzyme-based spectrophotometry is the most widely accepted method for detecting uric acid [5,6], and is an indirect measurement method. UA is first catalyzed by uricase to produce hydrogen peroxide, and then hydrogen peroxide is further oxidized into quinone imid pigment with the catalysis of catalase. UA detection is thus realized by monitoring the concentration of quinone imid pigment, as the spectral absorbance is correlated to the pigment concentration [5,7,8]. As the complex chemical reaction route is involved, interferences from other species and consequent selectivity problems become the primary concern; for example, interferences from ascorbic acid (AA). AA is a water-soluble vitamin and an essential inhibitor of scurvy [9]. As an interference with unsaturated bonds in its molecular structure in UA reaction, it oxidizes easily and depletes the hydrogen peroxide and benzoquinone sub, resulting in a false decrease in serum uric acid levels during uric acid determination [10,11,12]. Therefore, the elimination of AA hinders uric acid monitoring. Moreover, the vulnerability of enzymes and accompanying accuracy and stability problems also significantly impact the practical instrumentation attempt [13].

Spectroscopy detection involving no reaction and biomaterial may be a better solution for uric acid monitoring, and various pieces of research on different spectroscopy have been proposed [14,15]. As there is no chemical or biological reaction during spectroscopic detection, the critical points of a susceptible test are the ways in which to improve the interaction between electromagnetic waves and the analyte [16], as well as the selectivity accomplishment.

Metamaterials, artificial materials constructed from periodic cells that are subwavelength in size, provide a new strategy to enhance the wave–matter interaction [17]. Through the design of metamaterial structures, it is possible to obtain various electromagnetic properties not found in natural materials, including negative refractive index [18], electromagnetism-like induced transparency [19], and extreme environmental sensitivities [20]. The high sensitivity to small ecological changes is generally due to the surface plasmon polaritons (SPPs) [21] formed by the subwavelength periodic array. SPPs are abrupt waves propagating along the metal/dielectric interface. When the incident electromagnetic wave matches the oscillatory eigenfrequency of SPPs, surface plasmon resonance (SPR) is generated [22], manifested by a distinct resonance peak on the spectral line, which implies more energy coupling of the analyte and electromagnetic wave in this mode. As a result, trace analytes can be detected quantitatively, which means higher sensitivities and lower detection limits of the sensors. In biosensing, metamaterial sensors have gained increasing popularity.

As for the selectivity accomplishment part, Terahertz spectroscopy may be a practical option. Terahertz (THz) wave is an electromagnetic wave in the frequency range of 0.1–10 THz, which is located between microwave and infrared lights in the electromagnetic spectrum [23]. In addition to its electromagnetic properties, such as high penetration and low photon energy, it can provide fast responses that are non-contact and non-destructive [24]. More importantly, the rotation frequencies of the groups within many chemical or bio-molecules and the resonant frequencies of weak interactions between these molecules and the medium are primarily in the terahertz range, resulting in several distinctive fingerprint peaks for a certain analyte in the THz range [20], making THz detection useful in the field of biosensing. The selectivity of the target substance can be effectively enhanced by detecting metamaterials at the same resonant peak frequency as its characteristic peak frequency. This THz resonant coupling enhancement of the sensing effect has been found in many works. D. Lee et al. designed a metamaterial sensing enhanced by coupling with the characteristic mode of glucose to detect glucose and sucrose molecules [25]. Glucose showed a greater frequency shift than sucrose and transmittance was lower. Metamaterials with two kinds of steroid resonance modes were proposed and measured separately by S. Lee et al., with a significant drop in the transmittance of the target material [26]. The lactose characteristic pattern was applied to a device designed by Han et al. to detect lactose and fructose separately [27]. During THz biosensing, the metamaterial’s sensing process is based on resonant coupling between its plasma mode and molecular characteristic mode. It is important to note that the better the electromagnetic response pattern matches between them, the more molecular information is carried by the THz wave. By enhancing the sensing effect of THz resonant coupling, the target substance can be detected selectively. As inspired by these ideas, selectivity problems for spectroscopic sensors may be solved by modulating the resonance peak to approach the eigenfrequency of the target.

In this work, we propose a spectroscopic THz metamaterial UA sensor without any modifications nor any enzyme-like biomaterial. A theoretical model of this UA sensor was developed using a finite element simulation method and the simulation sensitivity was calculated to be 805.5 GHz/RIU. Both the sensitivity and selectivity of the UA sensor were demonstrated in UA detection. The UA was detected with a sensitivity of 0.117 GHz/μM and the limit of detection (LOD) is 9 μM. The AA response was experimentally revealed and analyzed, as the main interference for the UA detection. High immunity to AA interference was demonstrated experimentally in the detection of hybrid solutions. The reliability of the sensing experiments is also verified. The possible principle is also proposed and discussed.

## 2. Principle and Design

### 2.1. Metallic Hole Array Metamaterial Sensing Model

Metallic hole array (MHA), a microstructure that penetrates subwavelength size holes in a metal plate, is a branch of metamaterials. MHA’s transmission properties in the visible wavelength band have long been researched. At a specific aperture size, a high transmittance peak can be obtained from MHA, with peaks that exceed predictions from previous small-aperture diffraction theory. This is because of the MHA’s surface plasmon resonance (SPR) mode being excited. This unusual light transmission phenomenon is known as extraordinary of transmission (EOT). According to the central wavelength equation of the EOT peak [28], an equation describing peak frequency shifts due to changes in the dielectric constant is transformed as below:

(1) $$\frac{d}{d\varepsilon_{2}}f_{c}=\frac{k}{a_{0}}{\left(\varepsilon_{2}\sqrt{\varepsilon_{2}{}^{2}+\varepsilon_{1}\varepsilon_{2}}\right)}^{-1}\;$$

where $f_{c}$ is the resonance frequency; k is a negative constant related to the electromagnetic vector direction; $a_{0}$ is the array period of MHA; and $\varepsilon_{1}$ and $\varepsilon_{2}$ are the MHA and ambient dielectric constants, respectively. A positive $\varepsilon_{2}$ indicates a negative derivative of the resonant frequency; thus, as the ambient dielectric constant increases, the peak moves to lower frequencies. With $\varepsilon_{2}$ around 1, a common value for medium, the derivative change is close to zero, which means that the shift in frequency should exhibit a good linearity with $\varepsilon_{2}$. As a result of these properties of the EOT, the MHA can be employed to quantify the analyte through resonant peak frequency shifts.

As multi-band optics research advances, researchers have discovered the EOT effect in the terahertz band as well. It is possible to obtain high transmission peaks in the THz band by modifying the size of the microstructure of MHA metamaterials. The simplicity of MHA metamaterials and the absence of dielectrics has made them an important direction of research in the design of terahertz metamaterial structures.

### 2.2. Metallic Slot Array Metamaterial Sensing Model

**Figure 1:**
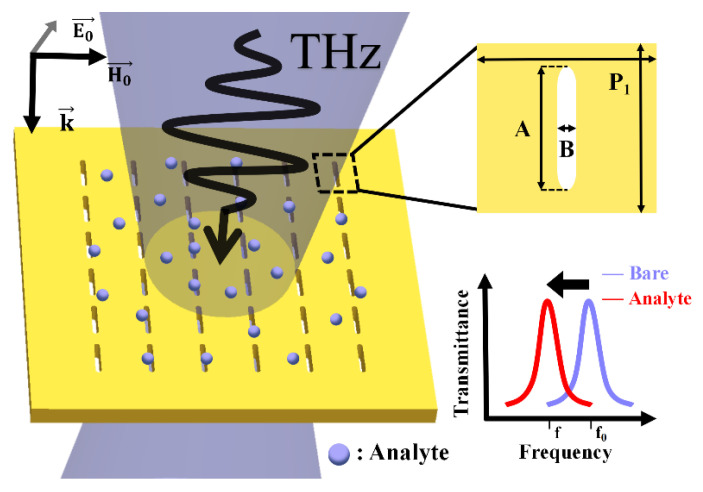
Schematic diagram of metallic slot array metamaterial biosensing.

Employing copper with low dielectric loss as a substrate, we designed a metallic slot array (MSA) metamaterial by compressing one diameter of a circular hole based on MHA. A significant THz-EOT phenomenon is also observed in the simulation. Figure 1 shows the sensing schematic of this MSA metamaterial sensor. When the analyte is deposited on the surface of the metamaterial, the dielectric constant of the environment will increase, i.e., $\varepsilon_{2}$ increases, resulting in a shift of the resonance peak to lower frequencies. By characterizing the amount of the center frequency shift, a quantitative measurement of the analyte can be achieved.

**Figure 2:**
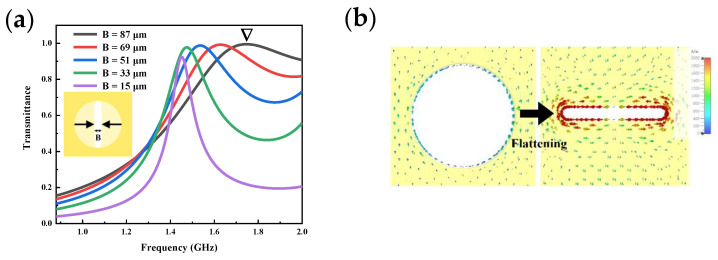

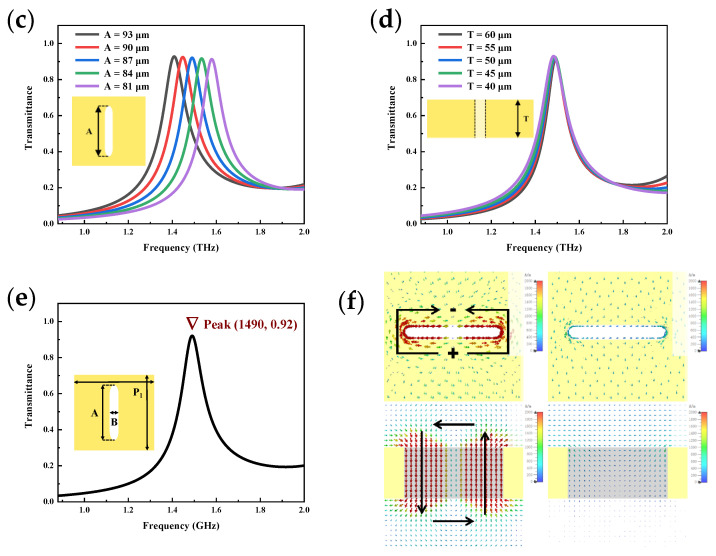
Optimization of MSA metamaterial cell sizes: (**a**) Optimization of slot width (B); (**b**) The variation in surface current after compressing; (**c**) Optimization of slot length (A); (**d**) Optimization of thickness; (**e**) Transmittance of MSA metamaterial simulations; (**f**) Surface currents (above) and magnetic fields (under) at resonant frequencies (1.49 THz) and non-resonant frequencies (0.88 THz).

To achieve THz resonance-enhanced sensing of UA, the resonance peak of the metamaterial needs to be modulated to coincide with the frequency of the characteristic peak of UA. The characteristic terahertz spectrum peak of UA is located at 1.5 THz [29], while AA is at 1.8 THz [30]. Metamaterial SPR resonant response modes are derived from their array structures by artificial design, so the array cell size of MSA metamaterial is significant. In our initial simulations, we observed an apparent EOT phenomenon in the circular hole structure at 0.88 THz-2 THz for a hole aperture of 87 μm. As shown by the black line in Figure 2a, the transmission spectrum exhibits a resonance peak with 99% transmittance, but a widened full width at half height (FWHM) at 1.7 THz. The quality factor (Q) defines the degree of energy loss in the system during one cycle, which is an important evaluation parameter correlated to the sensor performance. According to the calculation equation:

(2) $$Q=\frac{f_{0}}{\mathrm{FWHM}}$$

where $f_{0}$ is the center frequency of the resonance peak. Lower Q values imply higher energy loss in the system due to higher FWHM. According to Figure 2a, by compressing a hollow circular hole (B) into a hollow slot, the FWHM of the resonance peak at 1.7 T can be shortened and the Q value can be increased by 2.53 to 8.49.

A comparison and analysis of the surface currents in the slot cell and hole cell is shown in Figure 2b. Compared with the hole cell, the surface current formed by the THz incident wave on the slot cell has a higher energy density and is enriched at the tip of the slot, where the charge oscillation roots in a distinct electric dipole.

A similar THz photoelectric conversion effect was not found in the hole structure, which proves that the resonant coupling response of the THz wave and the SPP of the slot structure is stronger than that of the hole structure, resulting in a low energy utilization efficiency for incident THz waves. Figure 2c shows the transmittance curve change after changing the length of slot (A). The center frequency of the resonance peak shows a negative linear correlation with length of slot; when A is reduced from 93 μm to 81 μm, the center frequency is shifted toward a high frequency by 171.4 GHz. Accordingly, a solution to realize the resonance enhancement property of the metamaterial for UA sensing is proposed. The transmittance curve was also investigated in relation to substrate thickness. With the change in copper plate thickness, the resonance peak is not shifted much, as shown in Figure 2d. As a result, our MSA metamaterials are highly compatible with substrate sizes.

An MSA metamaterial with a resonance peak just coinciding with the UA characteristic peak is proposed, with the size optimization. The inset of Figure 2e shows the cell structure of the metamaterial. The cell period (P_1_) is 140 μm, the slot length (A) is 87 μm, the slot width (B) is 15 μm, and the thickness of the substrate is 50 μm. Figure 2g shows the transmittance curve of the metamaterial in 0.88 THz–2 THz. There is an apparent THz-EOT resonance peak about 92% transmittance at 1.49 T, with a Q value of 8.49.

**Figure 3:**
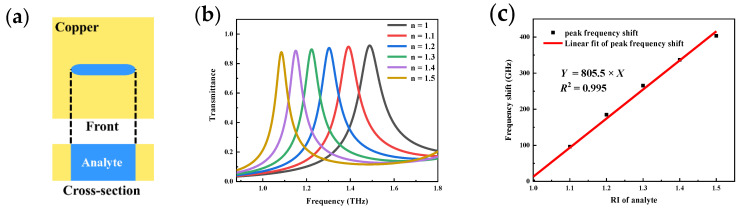
Simulation of metamaterial refractive index sensing performance: (**a**) Analyte filling situation; (**b**) Transmittance curve variation; (**c**) Peak frequency shift curve.

To investigate the formation principle of this resonance peak, the surface current (top) and magnetic field (bottom) of the metamaterial at resonant frequency (left, 1.49 THz) and non-resonant frequency (right, 0.88 THz) were simulated, as shown in Figure 2f. The incident THz wave generates charge oscillations at both ends of the slot under the EOT effect. It forms an electric dipole in the direction of the magnetic field. The current caused by the electric dipole flows backward along the ends of the slot on the metal surface. This results in a pair of magnetic dipoles in the hollow slot perpendicular to the face, but in opposite directions. Upon connecting them end to end, a toroidal dipole is formed. There is no effective charge oscillation at non-resonant frequencies, so a toroidal dipole is not formed in the hollow slot. EOT occurs primarily as a result of the coupling of electric and magnetic dipoles in this MSA metamaterial. The sensing character of MSA metamaterials towards dielectric environment is then simulated. Analyte with variable refractive index was filled into the slot in the simulation, as shown in Figure 3a. The thickness of the metamaterials is fixed at 50 μm. Transmission spectrums with gradient RI are shown in Figure 3b. A significant red shift occurs in the resonance peaks as the RI increases. The simulation sensitivity is expressed as the frequency shift generated by the unit RI change, which is calculated as

(3) $$S=\frac{\Delta f}{\Delta n}$$

where Δf is the frequency shift of the resonance peak and Δn is the change in RI. An excellent linear fit of the frequency shifts with the RI is found, as depicted in Figure 3c (R^2^ = 0.995). Based on Equation (3), the simulation sensitivity can be calculated as 805.5 GHz/RIU. The results demonstrate the high dielectric environment sensitivity of our proposed MSA metamaterial sensing model.

**Figure 4:**
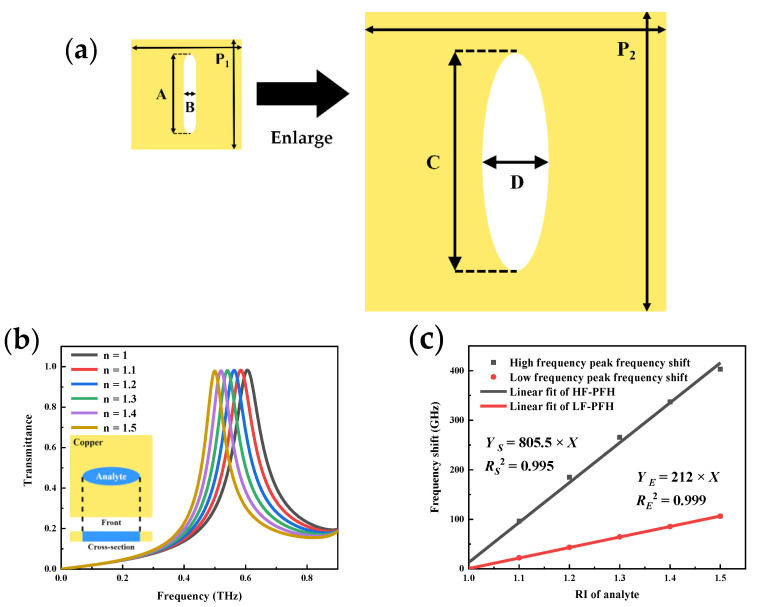
(**a**) Schematic diagram of low-frequency MSA metamaterial; (**b**) Simulated transmittance curves of different RI for the LF-MSA; (**c**) Comparison of the frequency shifts of the two resonance peaks.

Finally, an MSA metamaterial with a low-frequency (LF) resonance peak is created by scaling up the period and size of the cell to validate the MSA metamaterial sensing model. Figure 4a shows the schematic of the enlarged cell structure. The cell period (P_2_) is expanded to 400 μm, the slot length (C) is 280 μm, the slot width (D) is 90 μm, and the thickness is kept constant at 50 μm. The transmittance curve in Figure 4b, as the black line, shows that there is a resonance peak at 0.6 THz with a Q value of 3.55.

Figure 4b shows simulated analytes added to the slot to detect transmission spectral changes. The red line in Figure 4c shows the frequency shift fitting curve for LF-MSA metamaterial with a simulated sensitivity of 212 GHz/RIU. The black line shows the corresponding term for MSA metamaterial with a simulated sensitivity of 805.5 GHz/RIU, which is nearly four times higher than the former. This significant improvement in sensitivity can be attributed mainly to the increase in the resonance peak’s Q value. The validity of the MSA metamaterial sensing model is thus confirmed.

## 3. Processing and Experiment

### 3.1. Femtosecond Laser Processing

**Figure 5:**
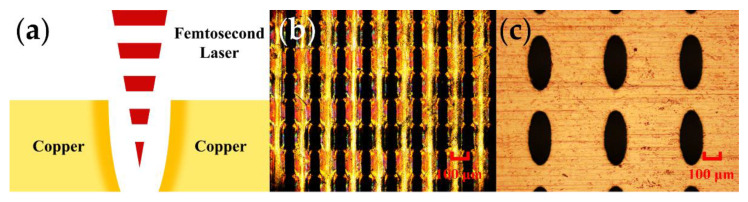
(**a**) Femtosecond laser processing schematic; (**b**) MSA metamaterial microscopy images; (**c**) Same size low-frequency MSA metamaterial microscopic images.

Femtosecond laser processing is employed to process the MSA metamaterial UA sensor. Figure 5a shows the schematic for femtosecond laser processing. In femtosecond laser processing, an ultra-short pulse laser with a high threshold effect ionizes the material directly. The metal can only be removed when the power density exceeds its ablation threshold. Thus, high-resolution machining accuracy can be achieved by modulation of the laser parameter, like pulse interval and output power, among others. In Figure 5b, a femtosecond laser-fabricated metamaterial UA sensor is shown microscopically. There is good agreement between the geometric design and the structural dimensions of the real metamaterial array. Figure 5c shows the microscopic image of the LF MSA metamaterial processed using the wet etching method, serving as an experimental counterpart.

### 3.2. Sample Preparation and Deposition Procedures

A series of sensing tests were performed for UA, AA, and a mixture of UA and AA to verify the usability and anti-interference to AA of this THz UA sensor. The UA and AA powders used in the experiments were purchased from (Aladdin, Shanghai, China). In normal human serum, UA levels range between 100 and 420 μM, blood UA levels exceeding 420 μM is the diagnostic criteria for hyperuricemia, and the highest concentration is about 600 μM for clinical observation. Detection ranges covering 100 to 650 μM are the standard technical requirement for practical devices.

A gradient concentrations of UA solution at 180, 340, 500, and 640 μM were prepared and tested. AA with the same gradient concentrations were also tested to verify the selectivity of this sensor for uric acid. Four other derivatives, dopamine, glucose, purine, and urea at 640 μM, were detected for comparison with the results in UA and AA assays. In addition, mixing 640 μM AA with four gradient concentrations of UA separately, a mixture for testing sensor resistance to AA interference was formulated.

**Figure 6:**
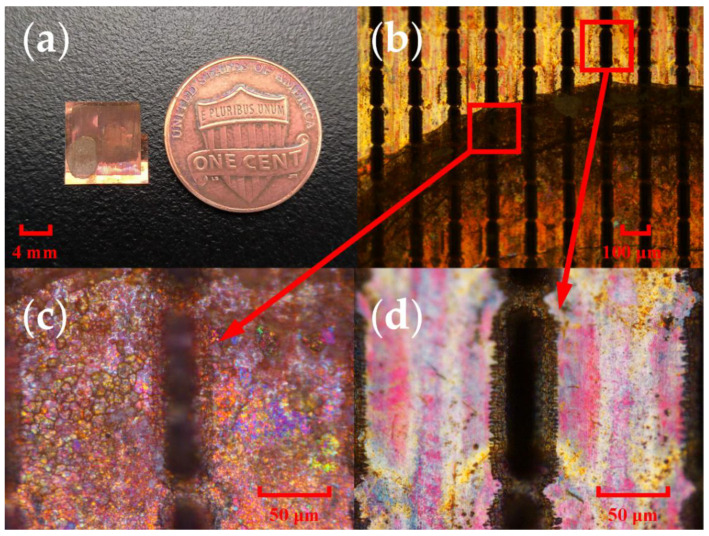
Sample transfer images: (**a**) Metamaterials after deposition of UA; (**b**) Edge of deposition area; (**c**) Slot unit after deposition; (**d**) Slot unit in clear area.

Figure 6a shows the picture of the THz UA sensor after the addition of UA by liquid phase deposition. The dark circle on the metamaterial indicates the area where UA is being deposited. A pipette gun was used to transfer 150 μL of the analytical solution to the sensor surface each time, where the droplets would remain on the sensor surface because of the surface tension of water. Using an 80 °C heating table, the sensor was then heated. Analytes were deposited on the sensor surface as the water evaporated. Before the next concentration of the solution was added dropwise, the sensor was cleaned using an ultrasonic shaker in deionized water to ensure that the deposits from the previous operation were eliminated. Figure 6b shows a microscopic image of the MSA metamaterial after the deposition of UA. The dark area below is the area of UA deposition, which differs significantly from the array above, where no UA is deposited. Figure 6c shows the hollow slot cell in the UA-deposited region in comparison with a non-deposited area, as shown in Figure 6d, indicating that UA has been added to the surface and the hollow slot.

Finally, for practical application scenarios, the long-term durability of the device and the humidity environment effect were investigated.

### 3.3. THz Spectral Measurement

**Figure 7:**
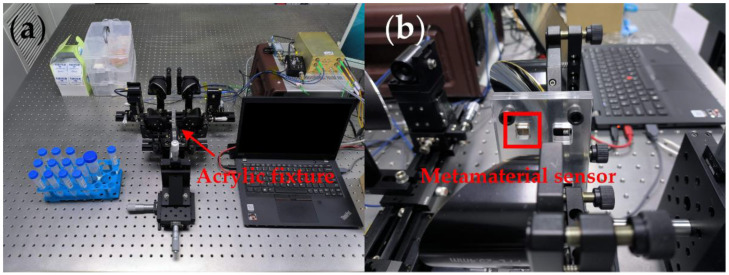
Spectral measurement images: (**a**) THz-FDS in sample measurement; (**b**) Sample fixing device.

All spectral data for this work were performed on the transmission test module of THz-FDS (Toptica terascan 1550, Munich, Germany). It mainly consists of two distributed feedback (DFB) lasers, two fiber-coupled InGaAs optical mixers, dual laser-controlled smart electronics, and a four-reflector transmission test optical path, as shown in Figure 7a. After deposition, the THz UA sensor will be placed in the THz-FDS for testing. THz waves are collimated by four 90° parabolic mirrors and focused on the UA sensor, which is fixed by a fixture mounted on an XYZ three-axis optical moving stage, as shown in Figure 7b. All transmission spectra between 0.88 THz and 1.65 THz were scanned in steps of 50 MHz with an integration time of 30 ms to obtain a more accurate spectral line.

## 4. Results and Discussion

A resonant peak with 96% transmittance in the 1.35 THz band was found, which can be used for THz resonance-enhanced sensing. Through the next experiments, we demonstrated that the THz MSA metamaterial UA sensor has a high sensing performance and selectivity for UA, which is well suited for future applications in the early monitoring and disease diagnosis of UA.

### 4.1. Frequency Shift Response in Different Sensors

**Figure 8:**
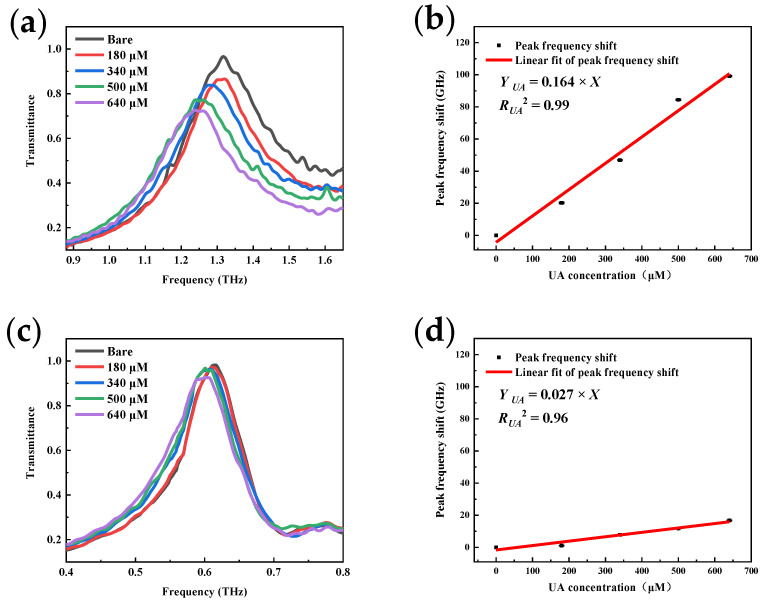
Detection results of different sensors with the same analyte: (**a**) HF peak shift with UA; (**b**) HF sensor calibration curve in UA detection; (**c**) LF peak shift with UA; (**d**) HF sensor calibration curve in UA detection.

Figure 8a illustrates the response of the resonance peak after deposition of a gradient concentration of UA solution on the HF sensor. There is a noticeable redshift phenomenon observed in the resonance peak, with a maximum frequency shift of 99.2 GHz and a gradient decrease in transmittance. These results agree with the results obtained from our simulated MSA metamaterial sensing model. The reproducibility of the sensing experiments was also verified by five repeatability tests for each concentration. According to the error bars on the calibration curves, the extremely low variance of the frequency shift proves the high stability of this sensor. All subsequent test data were obtained from the same stability tests. A Gaussian fit was used to determine the frequency shift due to the deposited UA. In Figure 8b, a good linear response is shown in the frequency shift of the resonance peak with an R^2^ of 0.99. Test sensitivity is determined as the frequency shift caused by a unit change in concentration. The sensitivity of the HF sensor in UA detection is 0.164 GHz/μM. In addition, HF sensor’s LOD for UA is calculated, which is 5.5 μM.

The LF sensor depositing the same gradient concentration of UA was also characterized. The transmittance curve variation of the LF sensor is shown in Figure 8c. A tiny concentration response is exhibited with a maximum shift of only 16 GHz. Figure 8d shows the results after linear fitting; in the same test situation, the tested sensitivity of the LF sensor is only 0.027 GHz/μM, which is one fifth of that of HF. This is very close to the results we obtained in the simulation of the MSA metamaterial sensing model and once again proves the correctness of our theoretical model. The sensing performance is obviously better with HF sensors, so all subsequent experiments were conducted based on HF sensors.

### 4.2. Frequency Shift Response for Different Analytes

**Figure 9:**
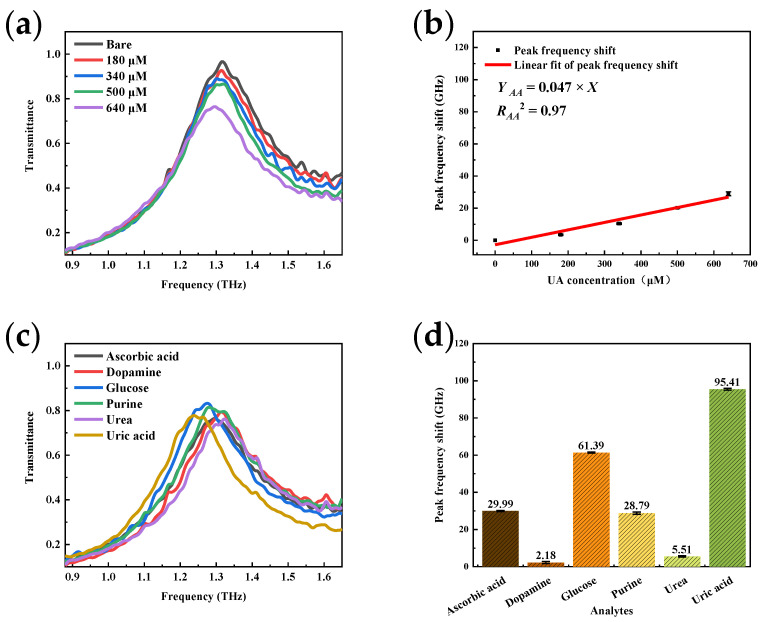
Detection results of the same sensor with different analytes: (**a**) Peak frequency shift with AA; (**b**) Sensor calibration curve in UA assay; (**c**) Corresponding response of different analytes; (**d**) Peak frequency shift of different analytes.

To validate the THz resonant coupled sensing enhancement theory, different analytes were deposited onto the sensor for transmission spectroscopy testing. In Figure 9a, the sensor’s spectral response diminishes when AA is deposited at the same gradient concentration, as a significant reduction in the resonance peak frequency shift compared with the UA result is observed. Compared with UA, owing to its characteristic fingerprint peak located at 1.8 THz, AA exhibits a worse coupling with the plasma mode of the sensor, and the redshift is thus reduced. The linear fit of the redshift is characterized as shown in Figure 9b. The test sensitivity is 0.047 GHz/μM, nearly a quarter of the UA, demonstrating sensor selectivity for UA. The LOD of the sensor for AA detection is 19.1 μM.

The resonant peak frequency shifts of different analytes at the same concentration (640 μM) were also measured. Figure 9c shows the serried transmittance curves, where different peak frequency shifts can be clearly observed. The central frequency shift after the Gaussian fit is characterized by Figure 9d. It can be clearly seen that uric acid and glucose, of which the characteristic peak couples better with the metamaterial resonance peak, show higher responses.

**Figure 10:**
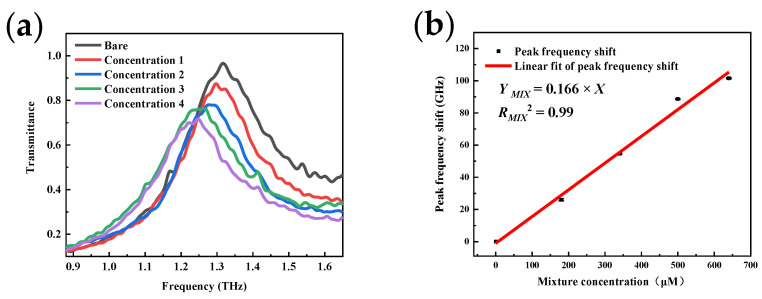
Detection results of the sensor’s resistant to ascorbic acid: (**a**) Peak frequency shift with the mixture of uric acid and ascorbic acid; (**b**) Sensor calibration curve in the mixture assay.

The mixture of gradient concentrations UA and 640 μM AA were measured to test the sensor’s resistance to AA in UA detection. The frequency shift of the resonance peak in Figure 10a is similar to that observed when only UA is deposited. According to Figure 10b, the slope of the linear fit curve is 0.166 GHz/μM, the same as that shown in Figure 8b.

Comparative analysis is also carried out on sensors proposed in other works, as shown in Table 1. UA is an important topic in blood analysis, but has rarely been mentioned experimentally in previous works, so several other results focused on different analytes are listed here. The linearity and sensitivity of the sensor output are the major concern for the metamaterial-based test. In works of Table 1, the sensitivity of the sensing is generally low, which is mainly because of the low Q value with the lower resonant peak frequency, just verifying the case of the LF sensor we measured. Poor coupling with the characteristic mode of the analyte is also a possible reason. The missing sensitivity of a part of the data is somehow attributed to the experimental errors and poor linearity of the test results, indicating the importance in modulating the sensing mode of the sensor.

All measured data are obtained from the transmissive metamaterial sensor in the THz band. The comparisons presented here are quite informative and show that our proposed device is capable of high sensing performance.

### 4.3. Sensor Test Stability

**Figure 11:**
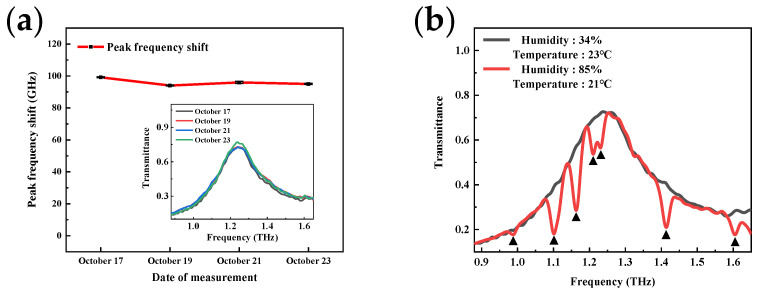
Sensing test stability characterization: (**a**) The long-term durability of fabricated sensors; (**b**) The effect of humidity environment on the device.

For real testing situations, the stability issue has always been an important factor. Figure 11a shows the sensor’s resonance peak frequency shift during one week in the detection of 640 μM UA. The inset is the THz spectral curves obtained from the metamaterial measured on October 17, 19, 21, and 23, respectively. The high durability was demonstrated by highly consistent results from four measurements at different times. The effect of a high humidity environment on the sensor is shown in Figure 11b. In the 640 μM uric acid assay, the humidity was increased to 85% by a humidity generator and the results were compared to those obtained from a normal environment. The red line is the high humidity result and the black line is the normal one. Owing to the strong absorption of terahertz by water, water vapor in air exhibits many absorption peaks in the terahertz band, and the peak frequencies in the range of 0.88–1.65 THz are 0.99, 1.1, 1.16, 1.21, 1.41, and 1.6 THz, corresponding to the dips marked in the red line. A high humidity environment will increase the absorption effect of these peaks, resulting a noise amplification. However, it is still quite cognoscible that the black line is the envelope of the red line, so it is feasible to attenuate or eliminate the interference caused by humidity through data post-processing to improve the stability of the testing.

## 5. Conclusions

In conclusion, a THz MSA metamaterial UA sensor is proposed and realized for enzyme-free detection of UA. The sensing theoretical model is validated in simulation and the fabrication is processed with femtosecond laser machining. Samples are characterized by a continuous wave THz-FDS. With the high Q factor and resonant frequency designed, high sensitivity and output linearity are found for UA detection. UA and AA can be effectively distinguished, as the sensor shows large variation in sensitivity. The test results of UA/AA mixture also demonstrate the resistance of the sensor to AA interference. This THz MSA metamaterial UA sensor has the advantages of small size, reusability, high detection sensitivity, low detection limit, and the capability to detect UA specifically, which is of great importance for the early detection and diagnosis of hyperuricemia in the future.

## Figures and Tables

**Table 1:** Comparison of metamaterial sensing performance.

Analytes	Sensitivity	Peak Shift/ Concentration	Frequency Range	References
Glucose	0.0122 GHz/μM 0.0052 GHz/μM	N/A	0.1 T–0.5 T	[31]
Glucose	N/A	67.5 GHz/3000 μM	0.3 T–0.6 T	[32]
Glucose	N/A	236.1GHz/19,000 μM	0.3 T–0.6 T	[33]
BSA	N/A	50 GHz/764.7 μM	0.72 T–0.9 T	[34]
BSA	0.097 GHz/μM	50 GHz/732 μM	1 T–1.7 T	[35]
Uric acid	0.164 GHz/μM	99.2 GHz/640 μM	0.88 T–1.65 T	This work

## Data Availability

Not applicable.
